# Concurrent Ludwig’s Angina and Agranulocytosis: A Two-Case Series Highlighting Diagnostic and Therapeutic Challenges

**DOI:** 10.7759/cureus.86679

**Published:** 2025-06-24

**Authors:** Pulkit Mehrotra, Steve Thomas, Prasanna S Kumar

**Affiliations:** 1 Medicine, Sri Ramachandra Institute of Higher Education and Research, Chennai, IND; 2 Haematology, Sri Ramachandra Institute of Higher Education and Research, Chennai, IND; 3 Otolaryngology, Sri Ramachandra Institute of Higher Education and Research, Chennai, IND

**Keywords:** agranulocytosis, airway obstruction, granulocyte colony-stimulating factor (g-csf), infection, ludwig’s angina

## Abstract

Ludwig’s angina (LA) is a rapidly progressive deep neck space infection that can become life-threatening due to airway compromise. Agranulocytosis, defined as a severe depletion of neutrophils, typically predisposes patients to overwhelming infections; however, its coexistence with LA is rare and poorly characterized. Here, we present two cases of patients with no classical predisposing factors who developed LA in the setting of agranulocytosis, each highlighting distinct clinical challenges in diagnosis and therapeutic decision-making.

Both patients responded well to the timely initiation of granulocyte colony-stimulating factor (G-CSF) and antimicrobial therapy, with complete resolution and neutrophil recovery by day 5-6. These cases reinforce the importance of routine hematologic monitoring in patients prescribed antithyroid drugs such as carbimazole and underscore the need for early clinical vigilance for deep neck infections in immunocompromised individuals.

## Introduction

Ludwig’s angina (LA) is a rapidly progressive cellulitis [1] involving the submandibular, sublingual, and submental spaces. The condition can lead to airway compromise due to tissue edema and infection. Agranulocytosis is characterized by a marked reduction in neutrophil count [2] and is commonly associated with drug reactions or bone marrow suppression. While both conditions are independently known to cause significant morbidity [3], their co-occurrence is rarely described.

The neutropenic state in agranulocytosis impairs mucosal immunity and phagocytic clearance, predisposing patients to polymicrobial infections, including those in anatomically vulnerable spaces such as the submandibular region. Disruption of oral barrier defenses and diminished neutrophil-mediated containment increase the risk of rapid local spread. This manuscript highlights two such cases and explores their clinical implications.

## Case presentation

Case 1

**Figure 1:**
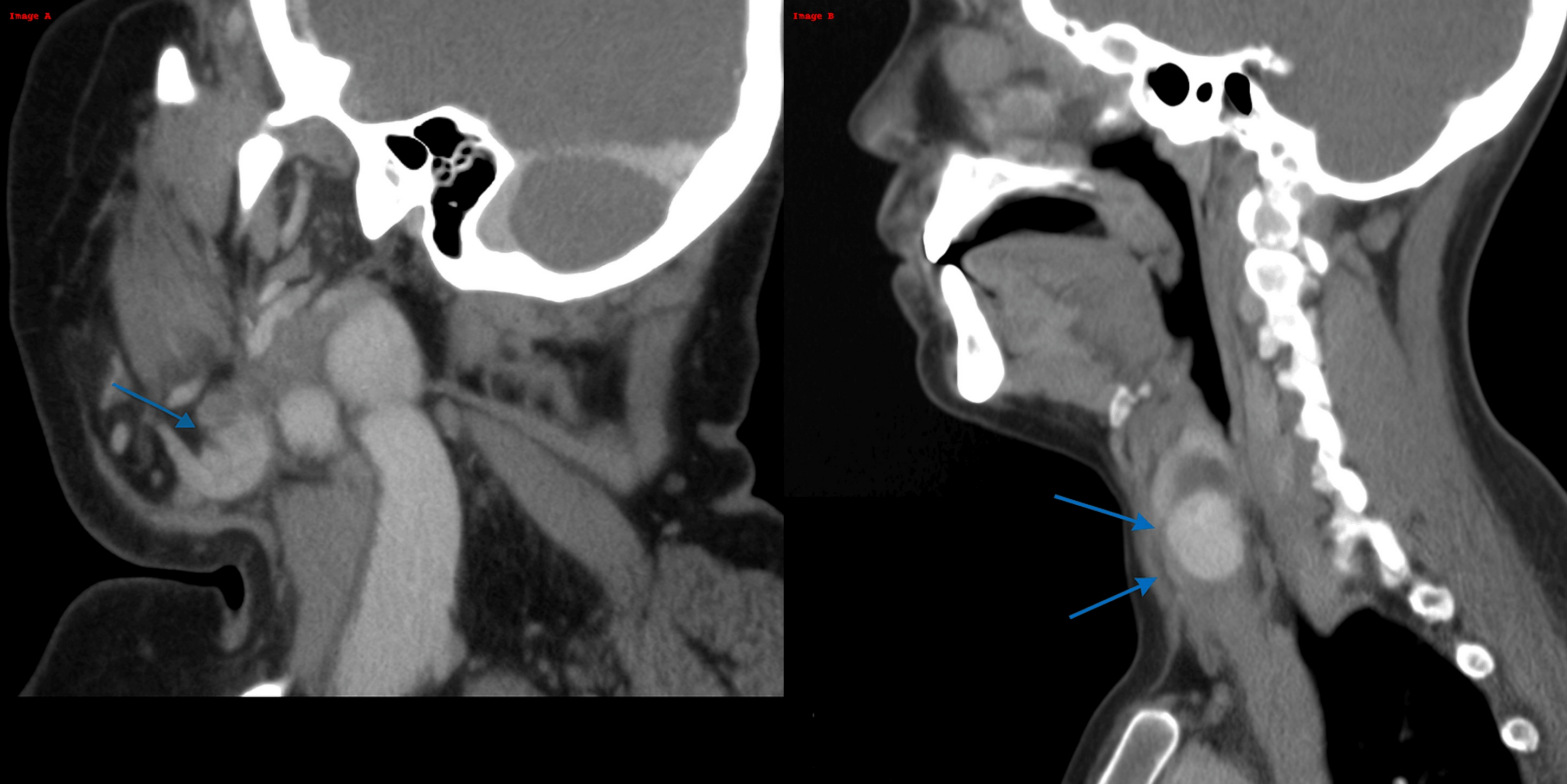
Contrast-enhanced sagittal CT showing a hypodense, rim-enhancing collection consistent with a retropharyngeal abscess. Image A: Contrast-enhanced sagittal CT image showing a well-defined, hypodense, rim-enhancing collection (blue arrows) in the anterior neck region, consistent with a retropharyngeal abscess. Image B: Higher sagittal CT slice demonstrating a similar hypodense lesion near the skull base and parapharyngeal space (blue arrow), raising concern for contiguous spread.

A 64-year-old woman presented with progressive submandibular swelling, fever (38.7 °C), and difficulty swallowing over three days. There was no history of dental trauma, oral sepsis, or recent procedures. Vitals showed mild tachypnea (RR: 22/min) and oxygen saturation of 95% on room air. Clinical examination revealed neck fullness, trismus, and restricted mouth opening. Her total leukocyte count was 510 cells/mm³ with an absolute neutrophil count <100 and normocytic anemia (Hb: 9.2 g/dL). A CT scan of the neck showed soft tissue stranding in the right submandibular space without fluid collection and an associated retropharyngeal abscess (Figure 1A). She was started empirically on IV piperacillin-tazobactam, metronidazole, and liposomal amphotericin B due to high risk of fungal superinfection in the setting of profound neutropenia. An ENT consult advised close monitoring without surgical drainage. Granulocyte colony-stimulating factor (G-CSF) was initiated on day 2, and the neutrophil count improved to >1,000 by day 6. No pathogens were isolated. She was discharged on day 9 with tapering steroids (used briefly for airway edema) and oral amoxicillin-clavulanic acid. Follow-up showed normalization of blood counts and complete resolution of swelling.

Case 2

**Figure 2:**
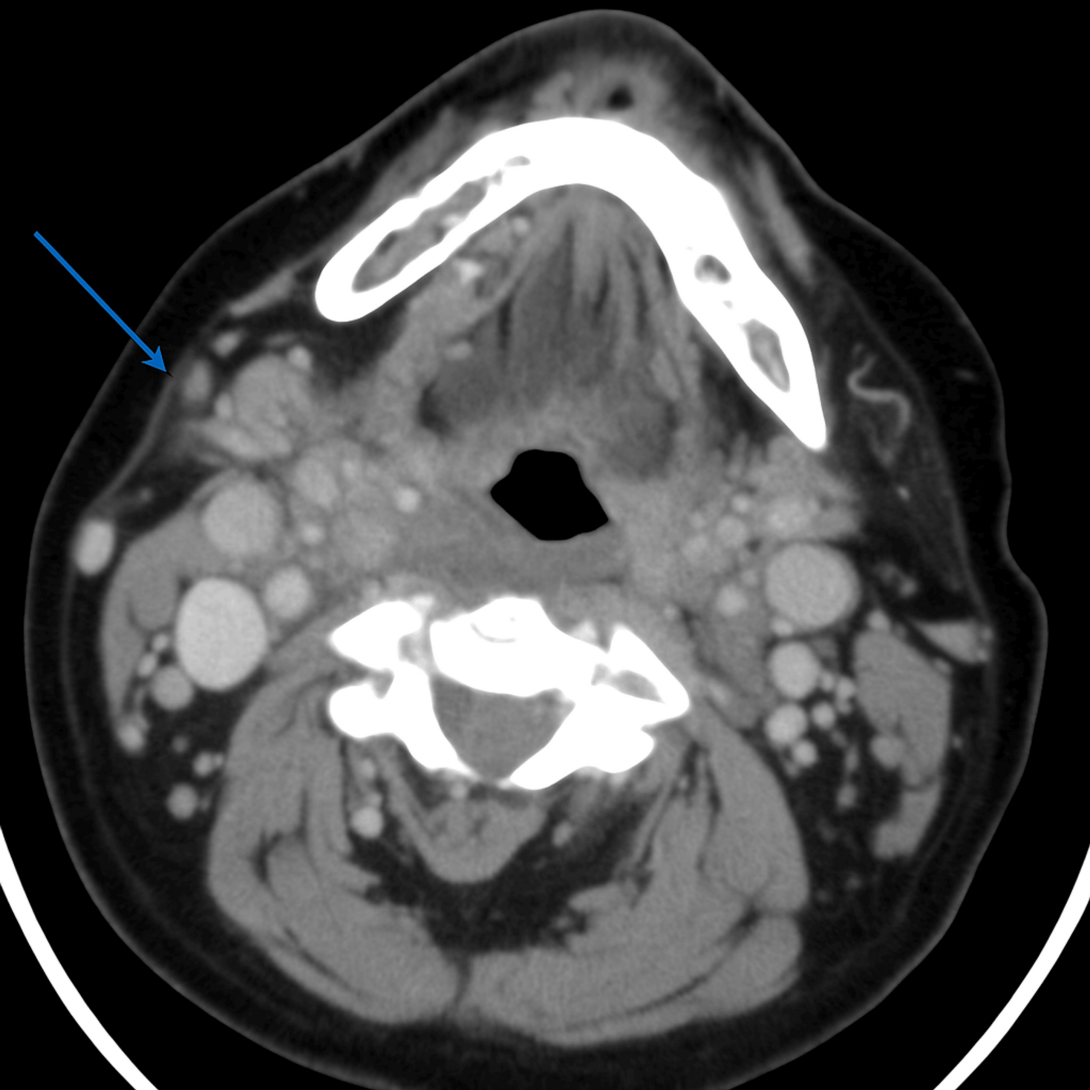
Axial contrast-enhanced CT scan showing minimal subcutaneous stranding at the right submandibular region (blue arrow), indicative of early inflammatory changes consistent with Ludwig's angina in an immunocompromised host.

A 58-year-old male with chronic obstructive pulmonary disease (COPD) presented with trismus, muffled voice, anterior neck swelling, and fever (38.4 °C) four days after starting carbimazole 30 mg/day for newly diagnosed hyperthyroidism. Baseline leukocyte count prior to carbimazole initiation was 6,400 cells/mm³. Vitals were stable except for mild tachycardia (HR: 102/min). Examination revealed tender submandibular induration and difficulty in phonation. Labs showed a total leukocyte count of 600 cells/mm³ with undetectable neutrophils, consistent with carbimazole-induced agranulocytosis. Thyroid-stimulating hormone (TSH) was suppressed, with elevated free T4. A CT scan revealed submandibular edema without abscess formation (Figure 2). Carbimazole was discontinued immediately. He was treated with IV meropenem and fluconazole, chosen for its favorable safety profile given the improving neutrophil trend and low suspicion for mold infections. G-CSF was given subcutaneously for three days. Neutrophil counts rose above 1,200 cells/mm³ by day 5. Throat swab and blood cultures were sterile. Clinical improvement began by day 4, and he was discharged on day 8. Endocrinology follow-up initiated alternative therapy for thyrotoxicosis post-discharge.

## Discussion

LA typically results from odontogenic infections. However, its manifestation in agranulocytosis, especially drug-induced, is exceedingly rare and often atypical [1,2]. Neutropenic states alter the host response to infection, blunting classic signs such as pus formation or erythema. This can delay diagnosis and understate radiologic findings [3,4].

In both cases, early imaging and recognition of subtle clinical clues (trismus, swelling, low oxygenation) helped establish the diagnosis. Importantly, the immunosuppressed state likely predisposed patients to deep tissue spread via impaired neutrophil recruitment and disrupted oral mucosal barriers [5].

G-CSF was initiated early, with neutrophil recovery within 5-6 days paralleling symptom resolution [6]. While G-CSF is generally safe, known risks such as bone pain or rare leukocytosis were monitored. Amphotericin B was given empirically in Case 1 due to high fungal risk (neutropenia, facial edema), despite the lack of fungal isolation. In Case 2, fluconazole was selected based on lower toxicity and clinical stability. Neither case required surgical drainage due to the absence of fluid collections on imaging and favorable immune recovery.

We identified two other published case reports of deep neck infections in neutropenic patients [7], supporting the rarity of this overlap. Our report adds to this literature by providing detailed hematologic, radiologic, and antimicrobial insight.

## Conclusions

This case series highlights a rare but clinically significant intersection between LA and agranulocytosis. Prompt recognition, individualized treatment, and early immune support (e.g., G-CSF) enabled successful non-surgical management in both cases. These cases underscore the importance of routine hematologic monitoring in patients started on antithyroid medications such as carbimazole and emphasize the need for early clinical vigilance for deep neck infections in immunocompromised individuals.
